# Premature Senescence and Telomere Shortening Induced by Oxidative Stress From Oxalate, Calcium Oxalate Monohydrate, and Urine From Patients With Calcium Oxalate Nephrolithiasis

**DOI:** 10.3389/fimmu.2021.696486

**Published:** 2021-10-21

**Authors:** Kamonchanok Chuenwisad, Pimkanya More-krong, Praween Tubsaeng, Nattida Chotechuang, Monpichar Srisa-Art, Robin James Storer, Chanchai Boonla

**Affiliations:** ^1^ Department of Biochemistry, Faculty of Medicine, Chulalongkorn University, Bangkok, Thailand; ^2^ Division of Urology, Mahasarakham Hospital, Mahasarakham, Thailand; ^3^ Department of Food Technology, Faculty of Science, Chulalongkorn University, Bangkok, Thailand; ^4^ Department of Chemistry, Faculty of Science, Chulalongkorn University, Bangkok, Thailand; ^5^ Office of Research Affairs, Faculty of Medicine, Chulalongkorn University, Bangkok, Thailand

**Keywords:** kidney calculi, oxalates, calcium oxalate, cellular senescence, oxidative stress, nephrolithiasis, urine

## Abstract

Oxidative stress, a well-known cause of stress-induced premature senescence (SIPS), is increased in patients with calcium oxalate (CaOx) kidney stones (KS). Oxalate and calcium oxalate monohydrate (COM) induce oxidative stress in renal tubular cells, but to our knowledge, their effect on SIPS has not yet been examined. Here, we examined whether oxalate, COM, or urine from patients with CaOx KS could induce SIPS and telomere shortening in human kidney (HK)-2 cells, a proximal tubular renal cell line. Urine from age- and sex-matched individuals without stones was used as a control. In sublethal amounts, H_2_O_2_, oxalate, COM, and urine from those with KS evoked oxidative stress in HK-2 cells, indicated by increased protein carbonyl content and decreased total antioxidant capacity, but urine from those without stones did not. The proportion of senescent HK-2 cells, as indicated by SA-βgal staining, increased after treatment with H_2_O_2_, oxalate, COM, and urine from those with KS. Expression of p16 was higher in HK-2 cells treated with H_2_O_2_, oxalate, COM, and urine from those with KS than it was in cells treated with urine from those without stones and untreated controls. p16 was upregulated in the SA-βgal positive cells. Relative telomere length was shorter in HK-2 cells treated with H_2_O_2_, oxalate, COM, and urine from those with KS than that in cells treated with urine from those without stones and untreated controls. Transcript expression of shelterin components (TRF1, TRF2 and POT1) was decreased in HK-2 cells treated with H_2_O_2_, oxalate, COM, and urine from those with KS, in which case the expression was highest. Urine from those without KS did not significantly alter TRF1, TRF2, and POT1 mRNA expression in HK-2 cells relative to untreated controls. In conclusion, oxalate, COM, and urine from patients with CaOx KS induced SIPS and telomere shortening in renal tubular cells. SIPS induced by a lithogenic milieu may result from upregulation of p16 and downregulation of shelterin components, specifically POT1, and might contribute, at least in part, to the development of CaOx KS.

## Introduction

Calculi or stones in the urinary tract form mostly in the kidneys, and the most common type is composed of calcium oxalate (CaOx) (1). The risk of forming kidney calculi gradually increases in the third decade of life and progressively declines in the seventh decade (2, 3). The peak age for forming a CaOx stone is between 40 and 60 years old (4–6). Nephrolithiasis has been documented since antiquity (7), but its pathogenesis remains far from full elucidation.

It is known that urinary ion saturation and consequent crystal formation are primary drivers of CaOx lithogenesis. Lithogenic substances including oxalate and CaOx crystals evoke oxidative stress in renal tubular cells through increased production of reactive oxygen species (ROS), activating inflammation (8). The ROS generated selectively activate the p38 mitogen-activated protein kinase (MAPK) pathway (9) that further activates inflammatory transcription factors, including nuclear factor (NF)-κB and transcription factor AP-1 (10, 11). Proinflammatory macrophages surrounding Randall’s plaques have been demonstrated in kidney tissue (11, 12). Oxidative stress, inflammation, and fibrosis have been clearly demonstrated in the kidney tissues of patients with nephrolithiasis (13–16). A proteomic analysis showed that inflammatory and fibrotic proteins are abundant in the urine and stone matrices of patients with nephrolithiasis (17). This evidence highlights that oxidative stress and inflammation are critically associated with the development CaOx calculi, and that CaOx nephrolithiasis could be considered as an inflammatory disease mediated by oxidative stress. Because age is associated with increased risk of CaOx stone formation and with cellular senescence (18), ROS may mediate the onset of cellular senescence (19), and when senescent cells accumulate, inflammation occurs (20–22). Therefore, we developed a hypothesis that cellular senescence is involved in CaOx lithogenesis. To our knowledge, no study has previously investigated whether cell senescence contributes to the development of kidney stone disease.

Senescence, a term coined by Hayflick and Moorhead in 1961 (23), is defined as an irreversible state of cell cycle arrest resistant to growth-promoting stimuli. However, senescent cells can remain metabolically active and accumulate with age, contributing to ageing and age-related diseases (18). Two main forms of cellular senescence have been established, replicative senescence and stress-induced premature senescence (SIPS). Replicative senescence is considered as natural cellular ageing resulting from an exhausted cell division (those reaching the so called “Hayflick limit”). The critically shortened telomeric length (genomic stress) activates a DNA damage response (DDR) and the p53-p21^CIP1^ growth-inhibitory pathway that further initiates replicative senescence (24). By contrast, SIPS, also known as stress or aberrant signaling-induced senescence (STASIS) or accelerated cellular senescence, designates a cell prematurely aged by chronic exposure to stressors at sublethal doses. SIPS is more likely than replicative senescence *in vivo* because cells are consistently exposed to stressors (both endogenous and exogenous) throughout life (24). Major recognized inducers of SIPS are oncogenic activation, oxidative stress, anticancer drugs, and radiation. SIPS stimuli that do not activate DDR or entail genomic damage will instead activate the cyclin dependent kinase inhibitor p16^INK4a^-pRB pathway to initiate senescence (25). Senescent cells acquire several features that distinguish them from other cells ranging from morphological changes (enlargement and flattening) to chromatin reorganization (26). Typical makers for cellular senescence include increased senescence-associated β-galactosidase (SA-βgal) activity, p16^INK4a^ upregulation, telomere attrition, and senescence-associated heterochromatic foci (26–28).

Physiological benefits of cellular senescence include its powerful tumor-suppressive mechanism that stops proliferation of premalignant cells (18, 21). Detrimental effects of senescence have also been recognized. Senescent cells acquire a senescence-associated secretory phenotype (SASP) that turns them into proinflammatory cells that actively produce and secret proinflammatory cytokines (21). In CaOx nephrolithiasis, oxalate and CaOx crystals induce oxidative stress and inflammatory cytokine release (10). Besides activating the p38 MAPK pathway *via* ROS, we speculate that cellular senescence might be an important link between oxidative stress and inflammation in CaOx lithogenesis. Oxalate and CaOx crystals may induce SIPS in renal tubular cells, and these premature senescent cells would produce SASP factors that amplify the inflammatory reaction.

Here, we investigated whether SIPS is induced after treatment of human kidney (HK)-2 proximal tubular cells with oxalate, calcium oxalate monohydrate (COM), and urine from patients with CaOx kidney stones (KS). Urine from individuals without stones (NS) served as a control. We determined SA-βgal activity, p16^INK4a^ expression, and telomere shortening. In addition, we also determined transcript expression of shelterin components including telomeric repeat binding factor 1 (TRF1), telomeric repeat binding factor 2 (TRF2), and protection of telomere 1 (POT1) in HK-2 cells exposed to CaOx lithogenic factors.

## Methods

### Cell Line and 24 h Urine Specimens

We used 24 h urine samples obtained from 10 participants as used for our previous study (29), 5 samples from patients with CaOx KS and 5 from age- and sex-matched individuals without stones (NS). The protocol using leftover specimens was approved by the Institutional Review Board of the Faculty of Medicine, Chulalongkorn University, Bangkok (IRB No. 642/63, COA No. 1189/2020). Demographic data for the donors and characteristics of the urine specimens are shown in **Table 1**.

**Table 1:** The characteristics of donors of 24-h urine samples used in the study.

Variable	Urine from patients with CaOx KS	Urine from people without (NS)	*P*
**n**	5	5	
**Male : Female**	4:1	4:1	
**Age (mean ± SD)**	58.4 ± 6.9	57.6 ± 6.2	0.852
**BMI (kg/m^2^)**	22.2 ± 3.4	23.0 ± 3.1	0.722
**24-h urine (mL)**	1,676 ± 403	1,862 ± 1,401	0.783
**Urine creatinine (g/day)**	1.77 ± 2.1	2.1 ± 0.5	0.738
**Urine oxalate^†^ (mg/day)**	18.6 ± 14.3	4.0 ± 1.3	0.015
**Urinary iCOCI^‡^ (COM equivalent, g/day; normal reference: < 0.8)**	3.8 ± 1.7	0.1 ± 0.3	0.002

^†^Measured by HPLC.

^‡^Measured by iCOCI (indole-reacted calcium oxalate crystallization index) method (29).

The HK-2 cell line (ATCC, CRL-2190) (30) was maintained in Dulbecco’s modified Eagle’s medium (HyClone Laboratories) containing 10% fetal bovine serum (HyClone) and 1% penicillin–streptomycin (GIBCO) at 37°C under an atmosphere of 5% CO_2_ and 95% humidity. Cells between their 65^th^ and 67^th^ passage were used in all experiments. The average diameter of HK-2 calculated across passages is 18.2 µm (31). HK-2 cells were treated with H_2_O_2_, sodium oxalate (NaOx) (Sigma-Aldrich), COM (Merck-Millipore), urine from patients with KS (n = 5), and urine from those without stones (NS urine) (n = 5) for 72 h. Cultures were treated at least in triplicate. According to their indole-reacted calcium oxalate crystallization index (iCOCI) levels (29), 24-h urine samples were pooled (from 10 samples), and divided into three groups, viz.: pooled urine with low or normal iCOCI levels from those without KS, pooled urine with high iCOCI from patients with KS, and pooled urine with very high iCOCI from patients with KS.

### Cell Viability

We examined the cytotoxicity of the treatments with an assay using the tetrazolium dye 3-(4,5-dimethylthiazol-2-yl)-2,5-diphenyltetrazolium bromide (MTT; Sigma-Aldrich). HK-2 cells were treated with 25 µM H_2_O_2_, 900 µM NaOx, 25 µg/cm^2^ COM, or 10% (v/v) urine from patients with KS in 6-well plates for 72 h. Untreated cells and cells treated with NS urine were used as controls. Subsequently, the cells were incubated with a solution of MTT (0.5% mg/mL) in the dark at 37°C under an atmosphere of 5% CO_2_ and 95% humidity for 2 h. Yellow MTT was reduced to purple formazan crystals in viable cells. Cells were washed twice with phosphate-buffered saline (PBS), and nuclei were labeled with the fluorescent stain 4′,6-diamidino-2-phenylindole (DAPI; Sigma-Aldrich). Purple viable cells with fluorescent blue nuclei were visualized and imaged using an EVOS FL Auto 2 Cell Imaging System (Thermo Fisher Scientific).

The selected concentrations of H_2_O_2_, NaOx, COM, and urine were considered as sublethal concentrations for senescence induction. Our dose-dependent experiments showed that concentrations higher than 25 µM H_2_O_2_, 900 µM NaOx, 25 µg/cm^2^ COM, and 10% (v/v) urine significantly decreased cell survival, and concentrations lower than these were not able to induce cell senescence significantly compared with untreated controls.

### SA-βgal Staining

We freshly prepared a staining solution of 50 mg/mL 5-bromo-4-chloro-3-indolyl-β-d-galactopyranoside (X-gal; Vivantis) in *N*,*N*-dimethylformamide (40 µL), 0.2 M citric acid–sodium phosphate buffer, pH 6 (1.74 mL), 200 mM potassium hexacyanoferrate (50 µL), 200 mM potassium ferrocyanide (50 µL), 5 M NaCl (100 µL), and 5 M MgCl_2_ (5 µL). Cells were fixed with 2% (v/v) formaldehyde and 0.2% (v/v) glutaraldehyde in PBS for 5 min and washed twice with PBS. The staining solution was added, and the mixture incubated at 37°C for 12–16 h. Stained cells were washed with PBS and imaged using an EVOS FL Auto 2 Cell Imaging System. The SA-βgal positive cells indicating senescence were stained blue.

### Protein Carbonyl Measurement

Proteins in treated cells were isolated using a radioimmuno-precipitation assay (RIPA) buffer containing a protease inhibitor cocktail (Thermo Scientific). The concentration of protein in cell lysate samples was determined using a bicinchoninic acid (BCA) Protein Assay Kit (Pierce). Protein carbonyl content, an indicator of protein oxidation, was measured using a 2,4-dinitrophenylhydrazine (DNPH) assay as described earlier (32, 33). In brief, cell lysates were incubated with 10 mM DNPH (TCI America) or 2 N HCl for 1 h in the dark, followed by incubation with trichloroacetic acid (20%) for 10 min on ice. Cell pellets were collected by centrifugation, washed with ethyl acetate:ethanol (1:1), and solubilized with 6 M guanidine hydrochloride (Sigma-Aldrich). Absorbance (A) was measured at 375 nm. The level of protein carbonyl (nmol/mg protein) was calculated from: ((A_DNPH_ – A_HCl_) × 45.45)/protein concentration.

### Total Antioxidant Capacity Determination

Total antioxidant capacity (TAC) was determined using 2,2′-azino-bis(3-ethylbenzothiazoline-6-sulfonic acid (ABTS; Sigma-Aldrich). In brief, ABTS radical cation solution (Sigma-Aldrich) was prepared and diluted to attain an absorbance (734 nm) of 0.65 ± 0.02. Sample or vitamin C standard or distilled water (blank) (5 µL) was added to the ABTS radical solution (295 µL) and incubated in the dark at 37°C for 10 min. Absorbance at 734 nm was measured again. Percent of antioxidant activity (%AA) was calculated from: ((A_blank_ – A_sample_)/A_blank_) × 100. A standard curve of %AA vs. vitamin C concentrations (0, 0.25, 0.5, and 1 mM) was created. TAC of sample was calculated from the standard curve and reported as vitamin C equivalent antioxidant capacity.

### Double Staining for SA-βgal and p16

Cells were grown on coverslips and stained for SA-βgal as described above. Immunocytofluorescence staining for p16 was performed subsequently. Cells were incubated with 10% Triton X-100 (Amreco) for 3 min and washed with PBS. Nonspecific binding was blocked by incubating with 1% normal horse serum (Gibco) at 37°C for 1 h. Cells were then incubated with anti-CDKN2A/p16INK4a antibody (Abcam, ab108349) (1:10,000) at 4°C overnight, followed by incubation with secondary antibody Alexa Fluor 488 conjugate goat anti-rabbit IgG (1:10,000) (Cell Signaling Technology) in the dark at 37°C for 30 min. The coverslip with stained cells was washed and mounted using Fluoroshield mounting medium and DAPI (Abcam, ab104139). SA-βgal and p16 positive cells were visualized and imaged using an EVOS FL Auto 2 Cell Imaging System.

### Western Blotting to Detect p16

We prepared 5% (stacking) and 12% (separating) polyacrylamide gels and loaded protein samples (10 µg) into wells in the stacking gel before electrophoresis at 100 V for 20 min, followed by 200 V for 1 h. Separated proteins were transferred to a polyvinylidene difluoride membrane, and nonspecific binding sites were blocked by incubating the membrane with 5% skim milk in Tris-buffered saline with Tween 20 for 1 h. The membrane containing the transferred proteins was incubated with anti-CDKN2A/p16INK4a antibody (Abcam, ab108349) (1:10,000) or rabbit anti-glyceraldehyde 3-phosphate dehydrogenase (GAPDH) monoclonal antibody (Cell Signaling Technology, catalog No. 5174) at 4°C overnight. Immunocomplexes were detected using ECL Western Blotting Substrate (Thermo Scientific) and visualized using a ChemiDoc MP Imaging System (Bio-Rad).

### Relative Telomere Length Measurement

Genomic DNA was extracted from cells using a GF-1 Tissue DNA Extraction Kit (Vivantis), according to the instructions from the manufacturer. The concentration of DNA was measured using a NanoDropTM 2000/2000c spectrophotometer (Thermo Fisher Scientific). DNA samples were stored at –20°C before real-time qPCR.

The relative telomere length (RTL) was determined by qPCR according to a procedure described previously (34, 35). Measurement of RTL was based on the ratio of copy number of telomeric repeat to copy number of a single-copy gene (*36B4*, encoding acid ribosomal phosphoprotein). This telomere to single-copy gene (T/S) ratio is proportionated to the average telomere length. The primers used were: telomere: forward (F) 5′-CGGTTTGTTTGGGTTTGGGTTTGGGTTTGGGTTTGGGTT-3′, telomere: reverse (R) 5′-GGCTTGCCTTACCCTTACCCTTACCCTTACCCTTACCCT-3′, 34B4: (F) 5′-CAGCAAGTGGGAAGGTGTAATCC-3′, and 36B4: (R) 5′-CCCATTCTATCATCAACGGGTACAA-3′. PCR was amplified at 95°C for 10 min, followed by 40 cycles of 95°C for 15 s, and 54°C for 1 min.

### TRF1, TRF2, and POT1 mRNA Expression

Total RNA was isolated from cells using a GF-1 Total RNA Extraction Kit (Vivantis), according to instructions from the manufacturer. The complementary DNA (cDNA) was converted from RNA templates using a TaqMan Reverse Transcription Kit (Thermo Scientific). qPCR (SYBR Master Mix, Biotechrabbit) for TRF1, TRF2, and POT1 was performed. The qPCR conditions were 95°C for 10 min, followed by 40 cycles of 95°C for 15 s, and 60°C for 20 s. The primers (36) were TRF1: (F) 5′-GCTGTTTGTATGGAAAATGGC-3′, (R) 5′-CCGCTGCCTTCATTAGAAAG-3′, TRF2: (F) 5′-GACCTTCCAGCAGAAGATGCT-3′, (R) 5′-GTTGGAGGATTCCGTAGCTG-3′, POT1: (F) 5′-TCAGATGTTATCTGTCAATCAGAACCT-3′, (R) 5′-TGTTGACATCTTTCTACCTCGTATAATGA-3′, and GAPDH: (F) 5′-AACGTGTCAGTGGTGGACCTG-3′, (R) 5′- AGTGGGTGTCGCTGTTGAAGT-3′.

### Statistical Analyses

Data are presented as mean ± standard deviation (SD). Difference between two conditions was determined using an unpaired *t* test. GraphPad Prism 9.2.0 was used to create graphs and for statistical calculations. Differences with *P* < 0.05 were considered significant.

## Results

### SIPS Induced by H_2_O_2_, Oxalate, COM, and KS Urine

**Figure 1:**
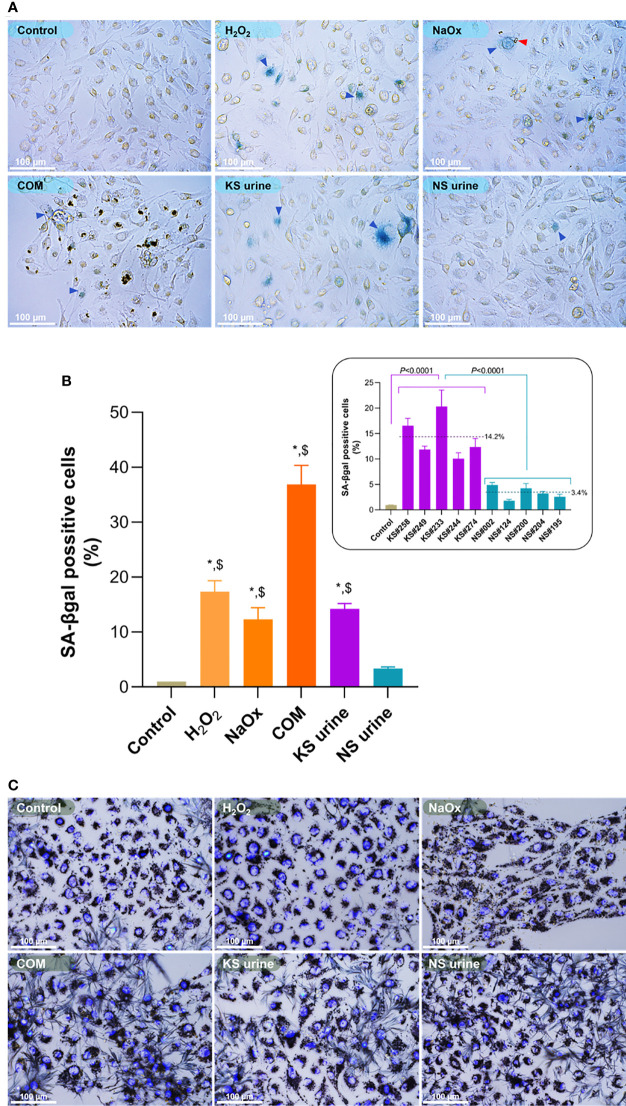
Induction of premature senescence by H_2_O_2_, sodium oxalate (NaOx), calcium oxalate monohydrate (COM), or urine from patients with kidney stones (KS). **(A)** SA-βgal staining in HK-2 cells treated for 72 h with H_2_O_2_ (25 µM), NaOx (900 µM), COM (25 µg/cm^2^), urine from patients with KS (10%, v/v) and those without KS (NS urine) (10%, v/v). More senescent cells (blue arrowheads) were observed in cultures of HK-2 cells treated with H_2_O_2_, NaOx, COM, and urine from patients with KS (KS urine) than in cultures treated with urine from those without KS or in untreated control cultures. The red arrowhead indicates CaOx formed after addition of NaOx. **(B)** The proportion of SA-βgal positive cells in cultures treated with H_2_O_2_, NaOx, COM, or urine from patients with KS were significantly higher than in untreated cultures or those treated with urine from donors without KS. Inset: proportion (%) of SA-βgal positive cells after treatment with individual samples of urine from patients with KS (KS samples) compared with treatment with individual samples from donors without KS (NS samples). Dashed line and number indicate mean. Error bars indicate SEM of at least triplicate treatments. **(C)** Cell viability assay showing treatment with H_2_O_2_, NaOx, COM, and urine at the concentrations used did not notably change the cell survival. Nuclei are stained by 4′,6-diamidino-2-phenylindole (DAPI). *P < 0.05 vs. Control, ^$^ *P* < 0.05 vs. NS urine. Magnification 400×.

The proportion of SA-βgal positive HK-2 cells was significantly greater after treatment with H_2_O_2_ (25 µM), NaOx (900 µM), or COM (25 µg/cm^2^) than the proportion in untreated controls (**Figures 1A, B**). The proportion of SA-βgal positive HK-2 cells was also significantly greater after treatment with KS urine (10%, v/v) than it was after treatment with urine from donors without KS (**Figure 1B**, inset). The proportion of SA-βgal positive HK-2 cells after treatment with urine from donors without stones was comparable to that in untreated control cultures (**Figures 1A, B**). Micrographs of SA-βgal staining after treatment with urine are shown in **Supplementary Figure 1**.

The cytotoxicity assay showed that treatment with H_2_O_2_, NaOx, COM, and urine at the concentrations used did not alter HK-2 cell survival significantly (**Figure 1C**). However, treatment with NaOx or COM tended to reduce the proportion of viable cells compared with other treatments. Representative micrographs showing viability of HK-2 cells treated with various concentrations (2.5%–40%, v/v) of urine are shown in **Supplementary Figure 2**.

### Oxidative Stress Induced by H_2_O_2_, Oxalate, COM, and KS Urine

**Figure 2:**
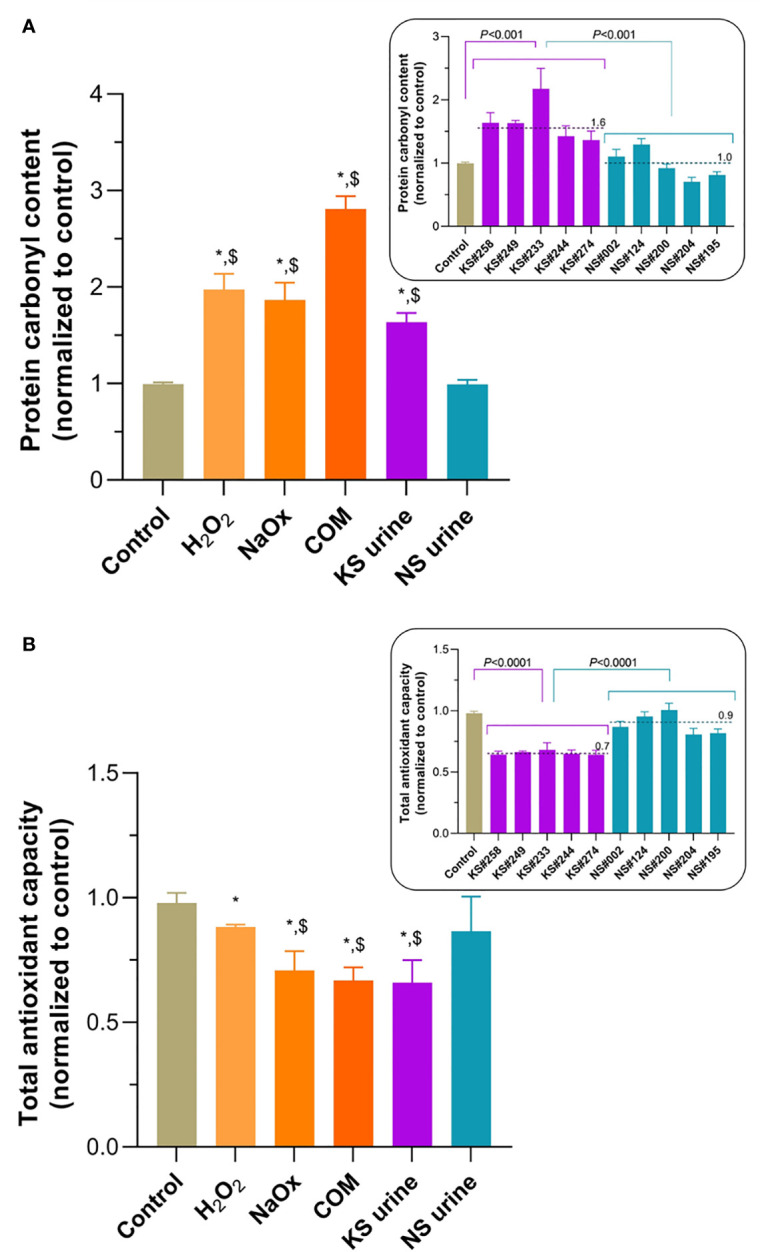
Oxidative stress induced by treatment of HK-2 cells with H_2_O_2_, sodium oxalate (NaOx), calcium oxalate monohydrate (COM), or urine from people with kidney stones (KS). **(A)** Levels of protein carbonylation after treatment with H_2_O_2_, NaOx, COM, or urine from patients with KS (KS urine) were significantly higher than those in untreated controls and after treatment with urine from patients without KS (NS urine). Inset: protein carbonyl level after treatment with individual samples of urine from patients with KS (KS samples) compared with treatment with individual samples from donors without KS (NS samples). **(B)** Total oxidant capacity (TAC) levels in HK-2 cells treated with H_2_O_2_, NaOx, COM, or urine from patients with KS were significantly lower than in untreated cells. TAC levels after treatment with NaOx, COM, and KS urine treatments were significantly lower than after treatment with NS urine. Inset: TAC level after treatment with individual samples of urine from patients with KS (KS samples) compared with treatment with individual samples from donors without KS (NS samples). Dashed line and number indicate mean. Error bars indicate SEM of at least triplicate treatments. *P < 0.05 vs. Control, ^$^ *P* < 0.05 vs. NS urine.

Protein carbonyl content of HK-2 cells treated with H_2_O_2_, NaOx, COM, or urine from patients with KS was significantly greater than that in untreated control cells or those treated with urine from donors without KS (**Figure 2A**). In every case, treatment with urine from patients with KS increased the protein carbonyl content of HK-2 cells significantly more than treatment with urine from donors without KS (**Figure 2A**, inset). Protein carbonylation levels in untreated cells and cells treated with urine from donors without KS were not significantly different. By contrast, TAC levels in HK-2 cells treated with H_2_O_2_, NaOx, COM, or urine from patients with KS were significantly lower than those in untreated controls (**Figure 2B**) or in cells treated with urine from donors without KS, which were not significantly different. Notably, TAC levels in HK-2 cells treated with urine from patients with KS (in all cases) were significantly lower than those in cells treated with urine from donors without KS (**Figure 2B**, inset).

### p16 Is Upregulated in Senescent Cells

**Figure 3:**
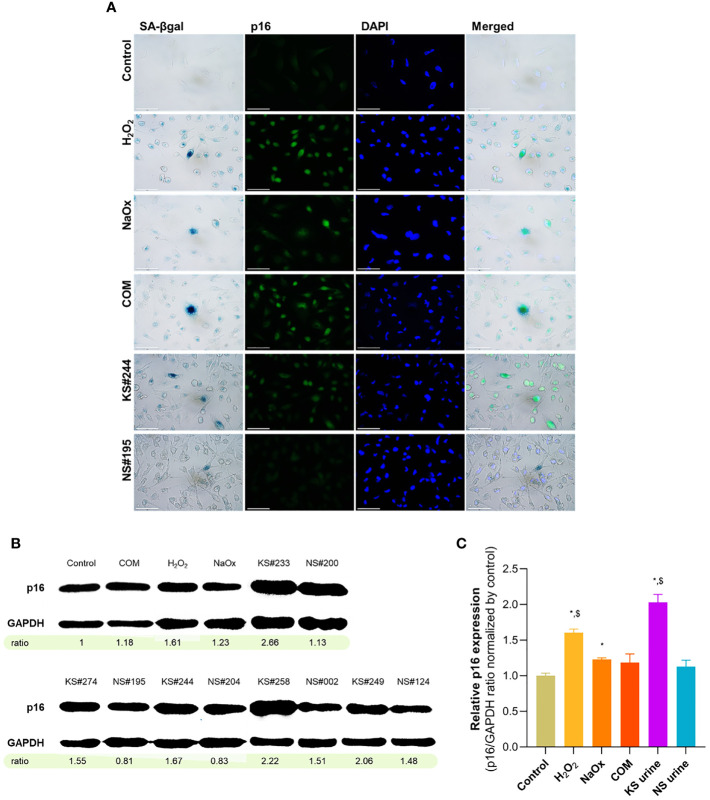
Upregulation of p16 in senescent HK-2 cells induced by their treatment with sodium oxalate (NaOx), calcium oxalate monohydrate (COM), and urine from patients with KS (KS urine). **(A)** Dual staining from SA-βgal and p16 immunofluorescence. p16 immunoreactivity is intensely positive in SA-βgal positive cells. **(B)** Western blot showing increased p16 expression in HK-2 treated with H_2_O_2_, oxalate, and urine from patients with KS. Numbers indicates intensity ratios of p16-to-GAPDH normalized by untreated control. **(C)** Relative p16 expression, indicated by intensity ratios of p16-to-GAPDH, compared between conditions. KS urine induced p16 expression significantly higher than NS urine and in untreated control cells. Error bars indicate SEM. Scale bar indicates 50 µm. *P < 0.05 vs. Control, ^$^ *P* < 0.05 vs. NS urine.

Substantial numbers of SA-βgal-positive cells were found after treatment of HK-2 cells with H_2_O_2_, NaOx, COM, and urine from patients with KS. p16 staining was intense in those SA-βgal positive cells (**Figure 3A**). Micrographs showing double-staining in cells after treatment with urine samples are shown in **Supplementary Figure 3**. The expression of p16 in HK-2 cell lysates was detected in western blots (**Figures 3B, C**). Although not as obvious as seen in the double staining experiment, p16 expression increased in HK-2 cells treated with H_2_O_2_, NaOx, and urine from patients with KS. Urine from patients with KS induced p16 expression significantly higher than urine from donors without KS and in untreated control cells.

Pooled urine from patients without KS did not induce SA-βgal-positive staining or upregulate p16, but the pooled urine with high iCOCI from patients with KS did (**Supplementary Figure 4**). Pooled urine with very high iCOCI levels induced apoptosis rather than senescence.

### Telomere Shortening Induced by H_2_O_2_, Oxalate, COM, and Urine From Patients With KS

**Figure 4:**
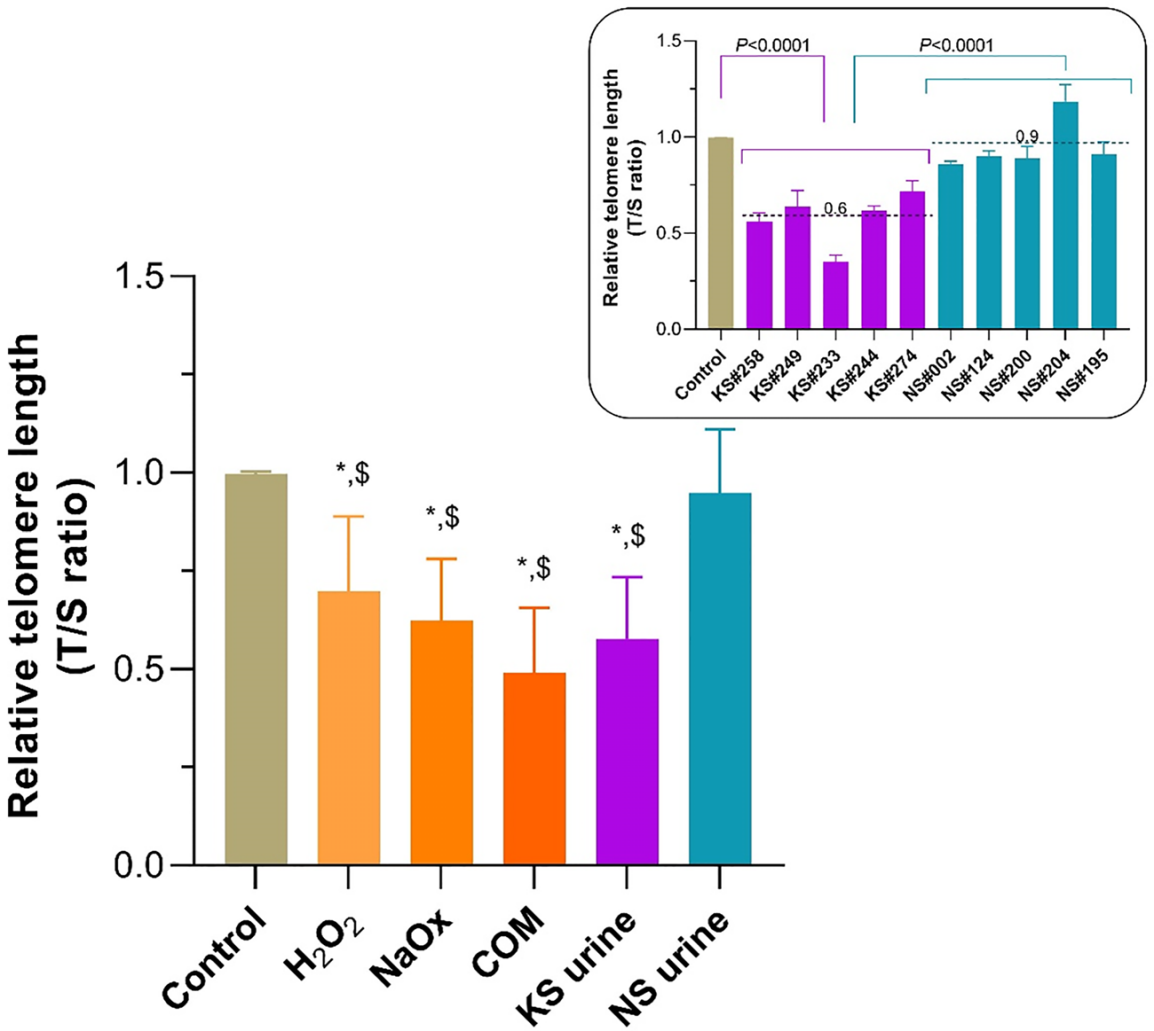
Shortening of telomeric length in HK-2 cells treated with sodium oxalate (NaOx), calcium oxalate monohydrate (COM), and urine from patients with KS (KS urine). The relative telomere length (RTL) was significantly reduced in HK-2 cells treated with NaOx, COM, or KS urine compared with the length in untreated control cells or those treated with urine from donors without KS (NS urine). In all instances KS urine reduced the RTL to almost half of that of the controls, and the extent of shortening was greater than after treatment with NS urine (inset). Dashed line and number indicate mean. Error bars indicate SEM of at least triplicate treatments. *P < 0.05 vs. Control, *^$^P* < 0.05 vs. NS urine.

RTL of HK-2 cells treated with H_2_O_2_, NaOx, COM, and urine from patients with KS was significantly shorter than it was in untreated controls (**Figure 4**). Treatment with urine from donors without KS did not alter the RTL compared with untreated control. Urine from patients with KS decreased telomere length by nearly half of that found in untreated controls. Every urine sample from patients with KS shortened telomeres more than urine from donors without KS did (**Figure 4**, inset).

### Urine From Patients With KS Reduced Expression of TRF1, TRF2, and POT1 mRNA

**Figure 5:**
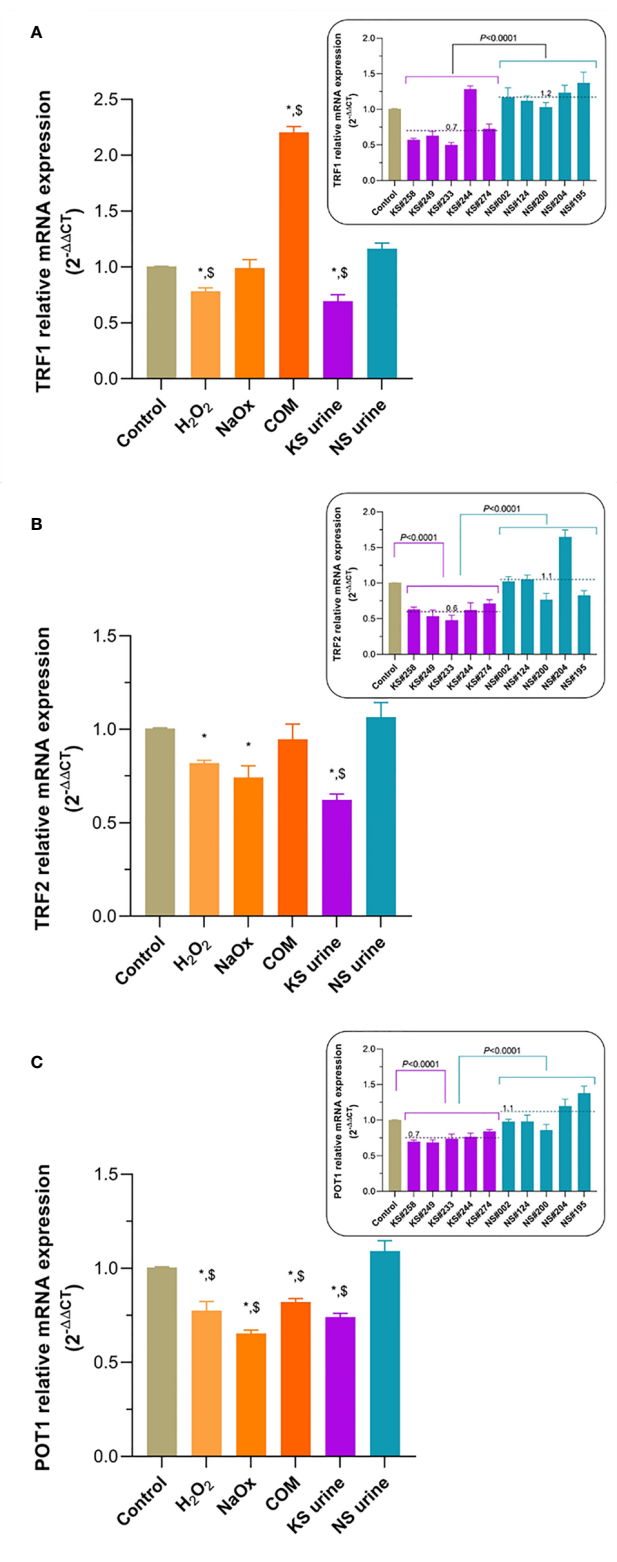
Transcript expression of TRF1, TRF2, and POT1 in HK-2 cells exposed to lithogenic factors. **(A)** TRF1 mRNA expression was significantly lower in cells after treatment with H_2_O_2_ or urine from patients with KS (KS urine), but significantly higher after treatment with calcium oxalate monohydrate (COM), compared with that in untreated controls or after treatment with urine from donors without KS (NS urine). **(B)** Treatment of HK-2 cells with H_2_O_2_ or NaOx significantly decreased TRF2 mRNA expression compared with that in untreated control cells, but KS urine treatment significantly decreased TRF2 mRNA expression compared with that in both untreated control cells and those treated with NS urine. **(C)** POT1 mRNA expression was significantly decreased in HK-2 cells treated with H_2_O_2_, NaOx, COM, and KS urine compared with that in untreated control cells or those treated with NS urine. Insets show expression of TRF1, TRF2, and POT1 mRNA after treatment with individual samples of urine from patients with KS (KS samples) compared with treatment with inidividual samples from donors without KS (NS samples). Dashed line and number indicate mean. Error bars indicate SEM of at least triplicate treatments. *P < 0.05 vs. Control, *^$^P* < 0.05 vs. NS urine.

TRF1 mRNA expression in HK-2 was significantly lower after treatment with H_2_O_2_ or urine from patients with KS, but higher after treatment with COM, than it was after treatment with urine from donors without KS or in untreated control cell (**Figure 5A**). TRF2 mRNA expression in HK-2 cells treated with H_2_O_2_, NaOx, or urine from patients with KS was significantly lower than it was in cells treated with urine from donors without KS or in untreated control cells (**Figure 5B**). POT1 mRNA expression was significantly lower in HK-2 cells treated with H_2_O_2_, NaOx, COM, or urine from patients with KS than it was in cells treated with urine from donors without KS or untreated control cells (**Figure 5C**).

## Discussion

We sought to determine whether premature senescence is involved in CaOx lithogenesis. Here, we show that treatment of HK-2 cells with oxalate, CaOx crystals, or urine from patients with CaOx KS induced oxidative stress, upregulated p16 expression, induced SIPS, and shortened telomeres. Senescent cells can enhance inflammatory reactions by producing and secreting SASP factors. Therefore, we consider that senescence could be a newly described mechanistic link between oxidative stress and inflammation in CaOx lithogenesis.

Oxalate and COM induce oxidative stress (8), oxidative stress triggers SIPS (37), and H_2_O_2_ induces SIPS through p16 upregulation (38). To our knowledge, this is the first report showing that lithogenic factors including oxalate, COM, and urine from patients with KS can induce the onset of SIPS in HK-2 cells, as indicated by increased SA-βgal activity and p16 upregulation. Notably, the upregulation of p16 was observed clearly in senescent cells. These findings suggest that the SIPS induced by oxalate, COM, and urine from patients with KS is mediated through oxidative stress and activation of the p16^INK4a^-pRB pathway.

We highlight that SIPS was only induced at sublethal concentrations of stressors (39). Extremely high concentrations of stressors induces a higher level of oxidative stress and apoptotic cell death (40). We found that treatment of HK-2 cells with concentrations higher than 25 µM H_2_O_2_, 900 µM NaOx, and 25 µg/cm^2^ COM, induced apoptosis instead of senescence. This lethal effect was extended to treatment with pooled urine with a very high iCOCI level, which induced apoptosis rather than senescence. The extent of oxidative stress depends largely on the level of ROS. Sies proposed the term “oxidative distress” for the high ROS level that deleteriously causes molecular damage and cell death, and an other term “oxidative eustress” for the lower ROS level that is beneficially essential in normal redox signaling (39). The ROS level that induces senescence is at the upper level of oxidative eustress. However, Bienertova-Vasku and Scheringer argued that both terms (eustress and distress) were rather the same, and suggested replacing them by “stress” alone (41). We prefer to retain “oxidative stress”.

In the present study, we found that treatment of HK-2 cells with H_2_O_2_, NaOx, COM, or urine from patients with KS increased oxidative stress and increased telomere shortening. Activating the signaling cascade in either the p53-p21^CIP1^ or the p16^INK4a^-pRB axis is required to initiate senescence (27). In replicative senescence, telomeres are shortened naturally, and senescence is signaled by activating DDR and p53-p21^CIP1^ pathways. By contrast, SIPS is prominently signaled by activating the p16^INK4a^-p38 MAPK pathway, and it is generally perceived that telomeres are not shortened significantly in SIPS (37). However, other evidence suggests that telomere shortening can be increased. Oxidative stress is well-characterized to increase telomere shortening (42–44). Essentially, DNA damage at the telomere is irreparable, because a shelterin complex blocks access by DNA repair proteins. When unrepaired oxidative DNA lesions accumulate at telomeric regions, telomere loss is inevitable (42). Single-strand breaks are the main telomeric DNA lesion causing shortening under oxidative stress (45). ROS not only attack telomeric DNA, but also nontelomeric DNA throughout the genome. Both telomeric and nontelomeric DNA lesions are formed under oxidative stress and can activate the DDR and p53-p21^CIP1^ pathways resulting in premature senescence (46). Although we did not measure telomeric DNA lesions, it is possible the lithogenic factors induced oxidative DNA lesions to form and accumulate at telomeres, which subsequently increased telomere shortening. Assessment of telomeric DNA damage and DDR activation are warranted in further study.

A so-called “end-protection problem” occurs when the ends of linear chromosomes or telomeres resemble the double-strand breaks that can activate the DDR pathways and then initiate repair processes (47). Mammalian telomeres use shelterin to solve this problem by forming a large lariat structure, the telomere loop (T-loop), to conceal the telomeric ends and protect them from being recognized by DDR components. Additionally, shelterin plays a role in regulating telomerase activity by recruiting telomerase to the telomere and driving the telomerase processivity (48). The shelterin complex consists of six protein components including TRF1, TRF2, Rap1 (bound to TRF2), TIN2, TPP1, and POT1. TRF1 and TRF2 bind to the double-stranded telomeric repeats. TRF1 facilitates telomere replication, while TRF2 facilitates T-loop formation. Both TRF1 and TRF2 help to recruit TIN2, TPP1, and POT1. By contrast, POT1 binds to the single-stranded TTAGGG repeats 3′-overhang. Oxidative stress induced telomere shortening and downregulation of TRF1, TRF2, and POT1 mRNAs has been demonstrated in human hepatocyte line L-02 (36). We showed that shelterin mRNAs, including TRF2 and POT1 are downregulated in HK-2 cells exposed to lithogenic factors. The level of POT1 mRNA was reduced the most. This implies that under lithogenic conditions formation of the shelterin complex in HK-2 cells is impaired. We speculate that this may result in less protection of telomeric ends and their greater exposure to the DDR system, with consequent increased telomere shortening.

The present study is limited because we did not assess oxidative telomeric DNA lesions or activation of DDR (ATM/ATR) or p53-p21^CIP1^ pathways. We did not measure expression of TRF1, TRF2, and POT1 proteins, nor telomerase activity. Whether senescent HK-2 cells generated under the lithogenic conditions can produce and secrete SASP factors remains to be determined. In addition to lithogenic factors, other substances existing in urine might also be able to induce premature senescence, but they were not investigated in this study. Mechanistic insight into the induction of premature senescence by lithogenic substances was not explored extensively in this study.

In conclusion, oxidative stress evoked by oxalate, CaOx crystals, and urine from patients with CaOx stones upregulated p16 expression, increased telomere shortening, and induced premature senescence in HK-2 renal tubular cells. Inducing SIPS appears to be mediated through cell senescence signaling pathways, p16^INK4a^-pRB and telomere shortening-DDR-p53-p21^CIP1^. Downregulation of shelterin components, especially POT1, may be responsible for the increased telomere shortening in HK-2 cells under lithogenic stress. Our data suggest that premature senescence induced by a lithogenic milieu is a newly described cellular mechanism that may, at least in part, contribute to CaOx lithogenesis.

## Data Availability Statement

The original contributions presented in the study are included in the article/**Supplementary Material**. Further inquiries can be directed to the corresponding author.

## Ethics Statement

The studies involving human participants were reviewed and approved by the Institutional Review Board of the Faculty of Medicine, Chulalongkorn University, Bangkok Thailand. The patients/donors provided their written informed consent to participate in this study.

## Author Contributions

CB contributed to the conception and design of the study, and analysis and interpretation of data for the manuscript. KC, PM-K, PT, NC, and MS-A contributed to acquisition of data. KC, NC, MS-A, and RJS contributed to its analysis or interpretation, or both. CB wrote the first draft of the manuscript. All authors contributed to revise the manuscript critically for important intellectual content, read and approved the version submitted for publication, and agree to be accountable for all aspects of the work with which they are associated.

## Funding

The study was supported financially by the Thailand Science Research and Innovation, Industry Division (grant No. RDG6150088 to CB), and the Ratchadaphiseksomphot Endowment Fund (grant No. GA64/51), Faculty of Medicine, Chulalongkorn University.

## Conflict of Interest

CB and NC are inventors of HydroZitLa, an antioxidant intervention for patients with kidney stones (patent pending). Chulalongkorn University and the inventors own the intellectual property for HydroZitLa.

The remaining authors declare that the research was conducted in the absence of any commercial or financial relationships that could be construed as a potential conflict of interest.

## Publisher’s Note

All claims expressed in this article are solely those of the authors and do not necessarily represent those of their affiliated organizations, or those of the publisher, the editors and the reviewers. Any product that may be evaluated in this article, or claim that may be made by its manufacturer, is not guaranteed or endorsed by the publisher.
