# Supplementary material for: Premature Senescence and Telomere Shortening Induced by Oxidative Stress From Oxalate, Calcium Oxalate Monohydrate, and Urine From Patients With Calcium Oxalate Nephrolithiasis
Source: Front Immunol. 2021 Oct 21;12:696486. doi: 10.3389/fimmu.2021.696486 (PMC8566732; doi:10.3389/fimmu.2021.696486)

Supplementary Materials

**Supplementary Figure 1** Representative micrographs of SA-βgal staining of HK-2 cells treated with urine from 5 patients with calcium oxalate (CaOx) kidney stones (KS) and 5 without (NS). A greater proportion of HK-2 cells stained with SA-βgal (blue) indicating senescence is seen after treatment with urine from patients with KS than after treatment with urine from those without stones. No SA-βgal positive cells or at least an exceptionally low proportion were found in untreated controls. Magnification 400×.


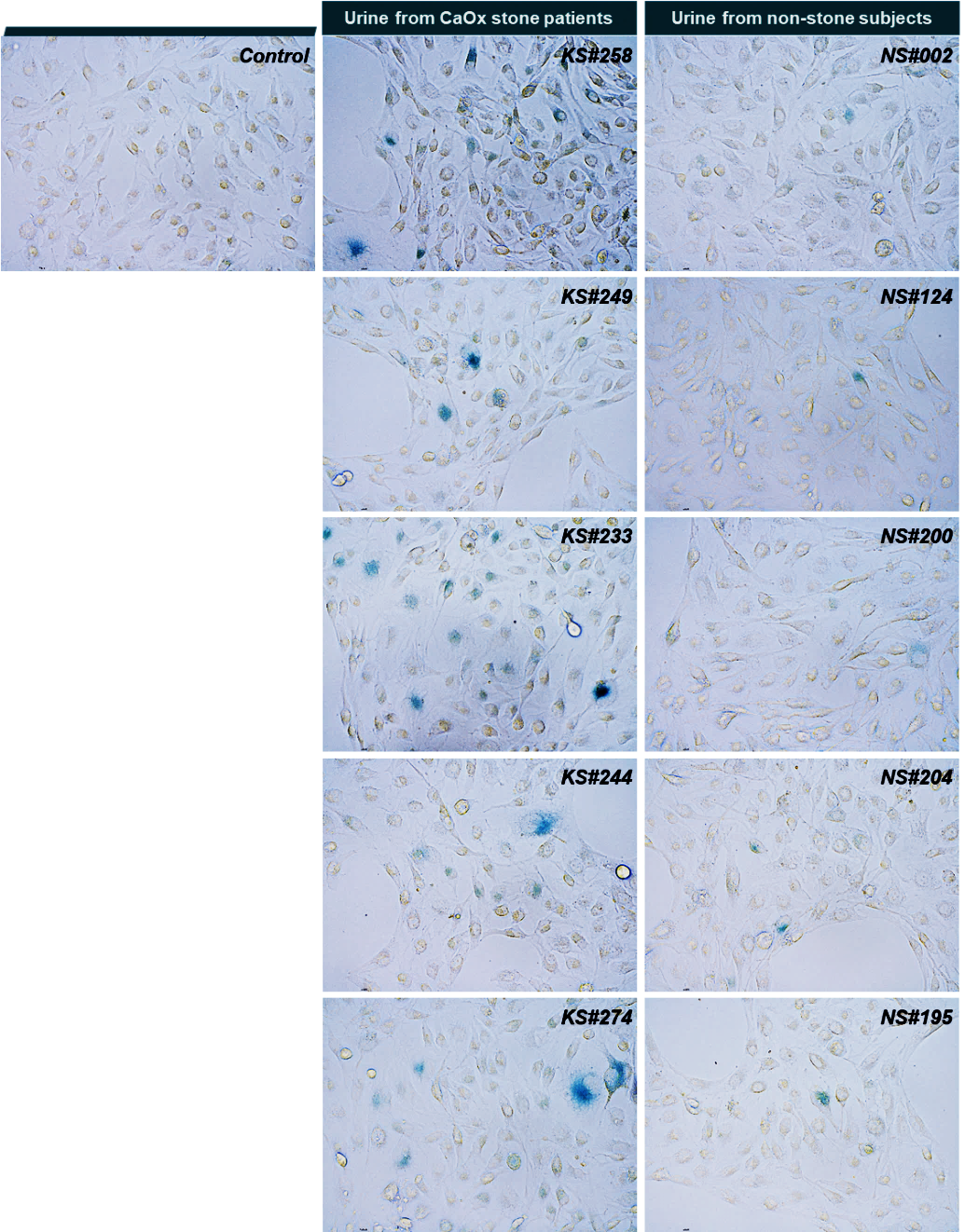


**Supplementary Figure 2** Representative micrographs of HK-2 cell viability assessed using an MTT assay after treatment with varied concentrations (2.5%–40%, v/v) of urine from patients with kidney stones (KS) or without (NS). KS#233 was the most toxic to HK-2 cells.


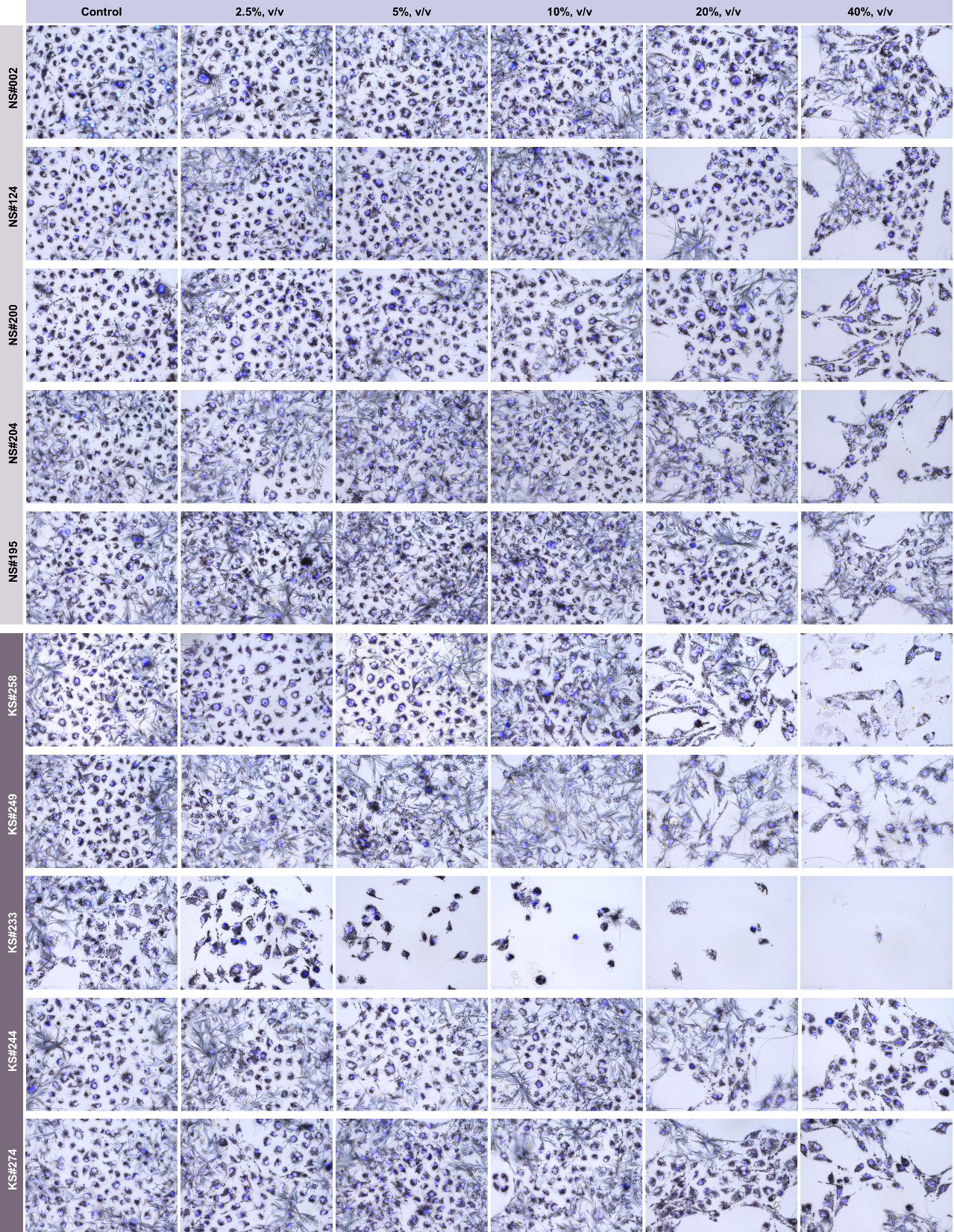


**Supplementary Figure 3** Double staining of SA-βgal and p16 immunofluorescence in HK-2 cells treated with urine from patients with kidney stones (KS) or without (NS) (A). Senescent cells highly express p16 protein, suggesting p16-mediated induction of senescence. KS urine markedly induced stress-induced premature senescence (SIPS) in HK-2, but NS urine did not. We did the experiment again (B) in three months later to see if the result could be reproduced, and the result was still the same.

**A.**


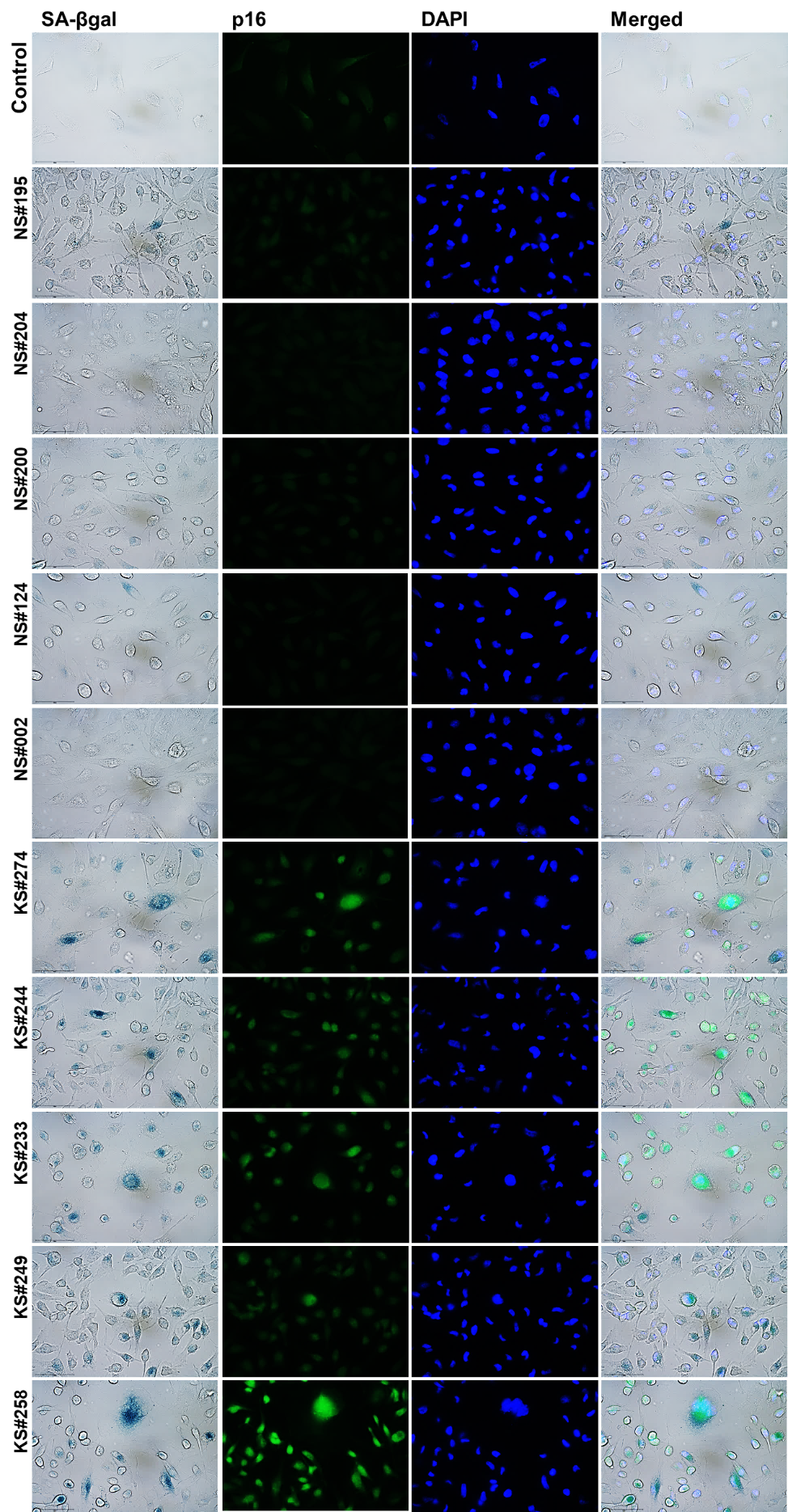


**B.**


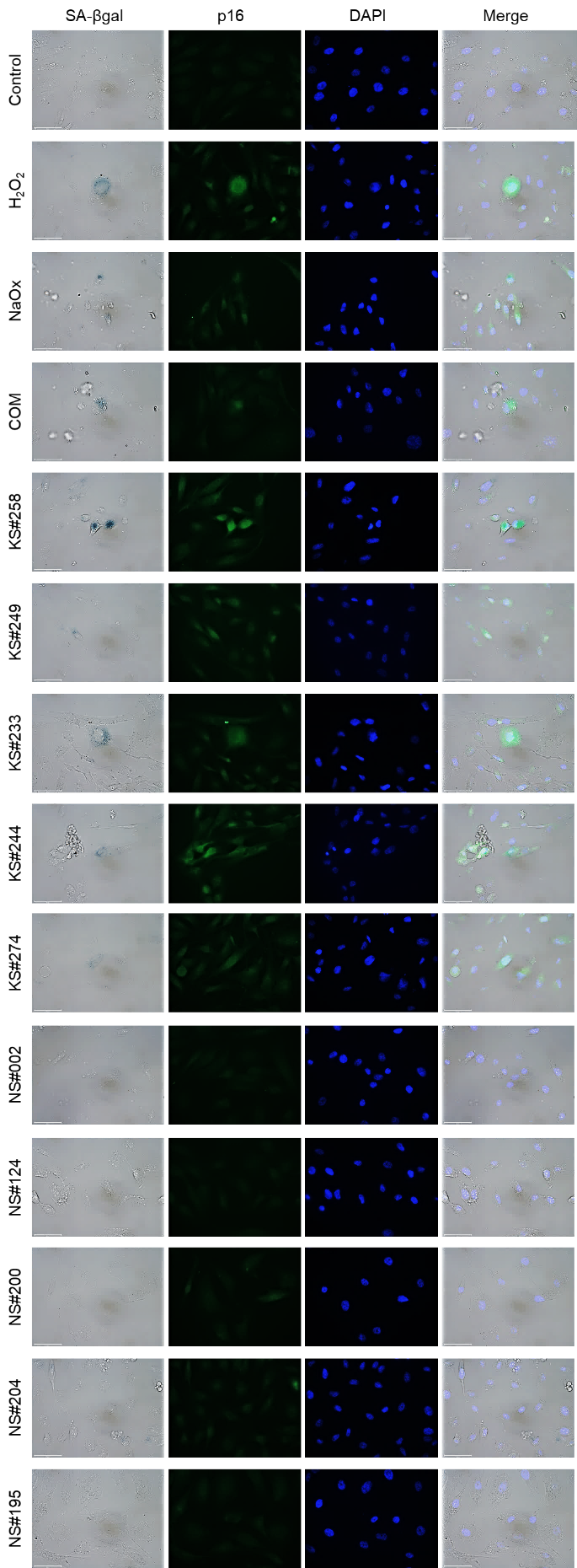


**Supplementary Figure 4** Induction of stress-induced premature senescence (SIPS) and upregulation of p16 in HK-2 cells treated with urine from patients with kidney stones (KS urine) or without (NS urine). Pooled urine samples from those with KS with high indole-reacted calcium oxalate crystallization index (iCOCI) induced SIPS (A) and upregulated p16 (B), but urine from those without stones (NS) did not. The urine with high iCOCI induced apoptosis rather than senescence. Western blotting showing increased expression of p16 in HK-2 cells treated with H_2_O_2_, calcium oxalate monohydrate (COM), and urine with high iCOCI (C). IF: immunofluorescence. Asterisks indicate SA-βgal positive cells.


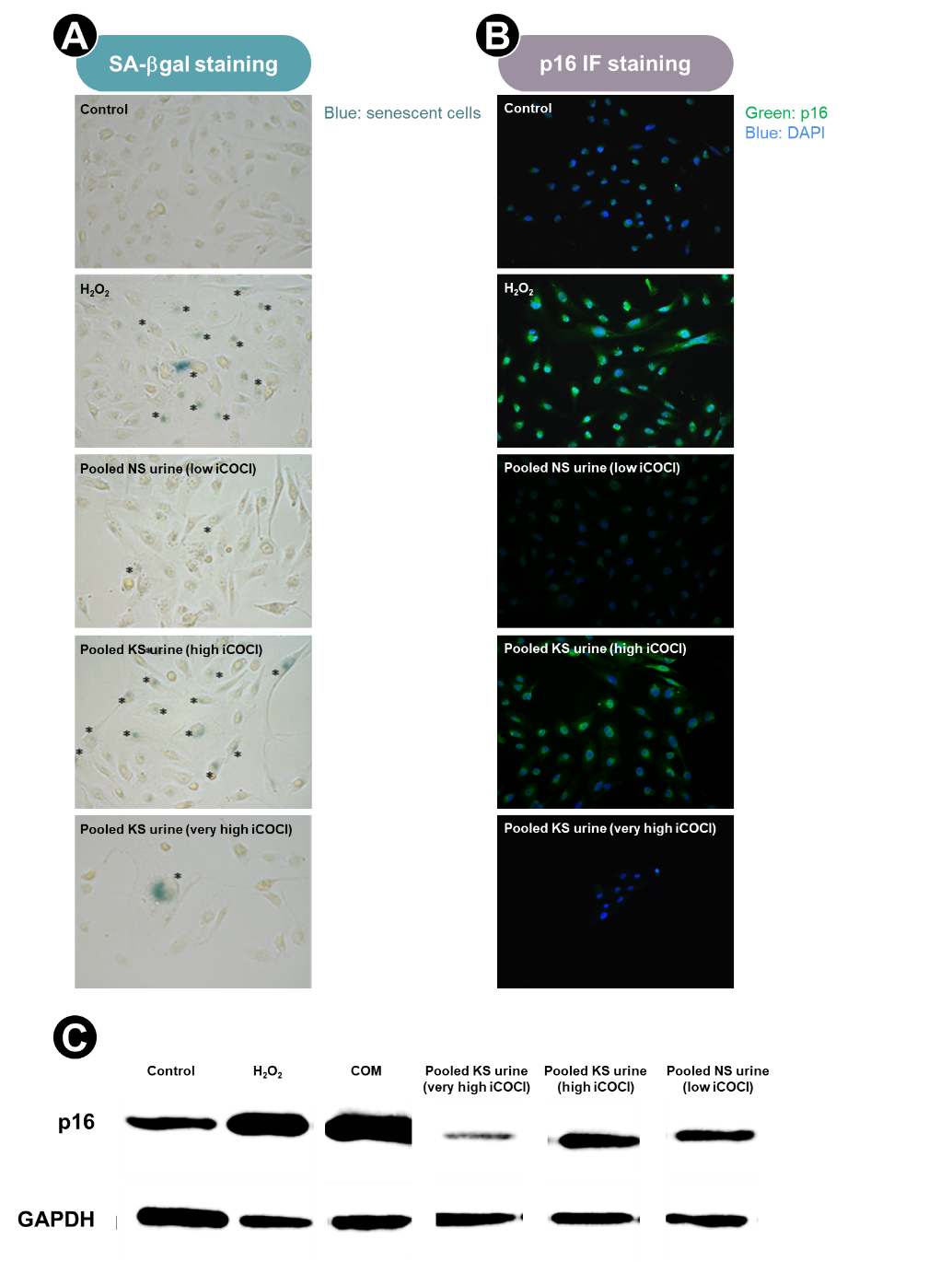

Supplement: Supplementary file 1 [file DataSheet_1.docx]
